# Supplementary material for: Longitudinal Position and Cancer Risk in the United States Revisited
Source: Cancer Res Commun. 2024 Feb 7;4(2):328–36. doi: 10.1158/2767-9764.CRC-23-0503 (PMC10848893; doi:10.1158/2767-9764.CRC-23-0503)
Supplement: Supplementary Table 1 — shows Types of Cancer Included in Composite Cancer Incidence Rate (19 total) [file crc-23-0503-s01.pdf]

Supplementary Table 1: Summary Statistics for Selected Variables

Statistic	Mean	St. Dev.	Min	Max
Cancer Incidence Rate (composite)	452.523	57.648	211.200	661.300
Relative Position	−4.091	4.557	−14.820	7.534
Below High School	0.133	0.062	0.011	0.467
High School	0.344	0.073	0.078	0.574
Some College	0.305	0.050	0.112	0.473
College and Above	0.218	0.096	0.032	0.753
Elevation (meters)	393.404	465.509	−21.000	3,163.000
Medical Doctor per capita	0.001	0.002	0.000	0.038
Median Income	48,579.740	13,163.530	21,087	220,645
Obesity Rate	33.722	5.876	11.000	58.900
Smoking Rate	21.340	4.240	7.076	40.937
PM2.5 (air pollution)	7.789	1.647	1.500	16.000
Water Violation	0.381	0.486	0	1
Race (White)	0.758	0.200	0.027	0.978
Race (Black)	0.095	0.146	0.000	0.859
Race (Native)	0.016	0.061	0.0004	0.892
Race (Asian)	0.014	0.025	0.000	0.384
Race (Hispanic)	0.098	0.140	0.006	0.964
Race (Other)	0.018	0.011	0.000	0.099

Number of Observations: 2853
