# Supplementary material for: Longitudinal Position and Cancer Risk in the United States Revisited
Source: Cancer Res Commun. 2024 Feb 7;4(2):328–36. doi: 10.1158/2767-9764.CRC-23-0503 (PMC10848893; doi:10.1158/2767-9764.CRC-23-0503)
Supplement: Supplementary Table 2 — shows sources for data. [file crc-23-0503-s02.pdf]

Supplementary Table 2: Data Sources

Data	Source
Cancer incidence rate	National Cancer Institute and Centers for Disease Control and Prevention
Educational Attainment	Economic Research Service, U.S. Department of Agriculture
Race	U.S. Census Bureau
Elevation	Open Elevation
Median income per capita	Bureau of Economic Analysis
Population Center	U.S. Census Bureau
Access to healthcare	Health Resources & Services Administration
Smoking rate & obesity rate, Air pollution & water violation	County Health Rankings & Roadmaps, University of Wisconsin Population Health Institute
