# Supplementary material for: Longitudinal Position and Cancer Risk in the United States Revisited
Source: Cancer Res Commun. 2024 Feb 7;4(2):328–36. doi: 10.1158/2767-9764.CRC-23-0503 (PMC10848893; doi:10.1158/2767-9764.CRC-23-0503)
Supplement: Supplementary Table 3 — shows summary statistics for selected variables [file crc-23-0503-s03.pdf]

Supplementary Table 3: Reported Coefficients of Relative Position for Hormonally Associated Cancers (with 95% Confidence Interval)

	Dependent variable:			
	Cancer Incidence Rate			
	Breast	Ovary	Prostate	Thyroid
	(1)	(2)	(3)	(4)
Relative Position	−0.285***	−0.006	0.387***	0.123**
	(-0.469,-0.101)	(-0.058,0.046)	(0.123,0.650)	(0.007,0.238)
Latitude	0.514***	−0.026	0.700***	0.090
	(0.165,0.862)	(-0.113,0.060)	(0.198,1.203)	(-0.106,0.286)
High School	45.179***	0.020	37.997**	21.493***
	(20.773,69.584)	(-6.808,6.848)	(2.708,73.286)	(5.815,37.171)
Some College	57.917***	−0.461	86.469***	7.868
	(37.167,78.666)	(-6.082,5.160)	(56.234,116.704)	(-5.232,20.967)
College and Above	82.081***	−2.537	71.577***	5.127
	(60.290,103.872)	(-8.185,3.111)	(39.726,103.428)	(-8.184,18.438)
Elevation	−0.006***	−0.0001	−0.005***	0.001*
	(-0.009,-0.003)	(-0.001,0.001)	(-0.009,-0.002)	(-0.0002,0.003)
Medical Doctor pc	4.735	−20.891	−322.516	−198.871**
	(-296.566,306.035)	(-88.867,47.085)	(-773.165,128.133)	(-356.383,-41.359)
Median Income	0.00004**	−0.00000	0.00004	0.00000
	(0.00000,0.0001)	(-0.00001,0.00000)	(-0.00001,0.0001)	(-0.00001,0.00002)
Obesity Rate	22.247***	−7.477***	15.190	−8.401*
	(8.124,36.369)	(-11.351,-3.603)	(-5.156,35.537)	(-17.135,0.333)
Smoking Rate	−105.899***	2.813	−104.505***	−32.751***
	(-144.468,-67.330)	(-6.480,12.106)	(-161.113,-47.896)	(-54.674,-10.829)
PM2.5 (air pollution)	0.138	0.110***	0.107	0.143*
	(-0.155,0.431)	(0.042,0.178)	(-0.319,0.533)	(-0.016,0.301)
Water Violation	1.408***	0.042	2.400***	−0.139
	(0.522,2.294)	(-0.163,0.246)	(1.119,3.681)	(-0.621,0.342)
Race (White)	−77.824**	9.754	209.432***	−20.454
	(-146.452,-9.195)	(-6.952,26.460)	(110.637,308.228)	(-58.808,17.900)
Race (Black)	−66.783*	9.113	288.464***	−29.607
	(-135.908,2.343)	(-7.731,25.958)	(188.715,388.212)	(-68.183,8.969)
Race (Native)	−105.620***	2.509	209.595***	−20.723
	(-178.470,-32.770)	(-15.283,20.300)	(104.050,315.139)	(-61.807,20.360)
Race (Asian)	−129.830***	10.906	157.325***	−13.694
	(-203.036,-56.624)	(-6.911,28.723)	(51.945,262.706)	(-54.732,27.343)
Race (Hispanic)	−89.638**	8.552	219.558***	−13.322
	(-158.906,-20.371)	(-8.331,25.435)	(119.763,319.353)	(-52.248,25.604)
Observations	2,615	890	2,623	1,191
R^2	0.534	0.276	0.573	0.504
Adjusted R^2	0.523	0.226	0.563	0.478
Residual Std. Error	0.388 (df = 2554)	0.286 (df = 831)	0.612 (df = 2562)	0.475 (df = 1132)
F Statistic	48.760*** (df = 60; 2554)	5.473*** (df = 58; 831)	57.412*** (df = 60; 2562)	19.825*** (df = 58; 1132)
Note:				*p<0.1; **p<0.05; ***p<0.01
