# Supplementary material for: Longitudinal Position and Cancer Risk in the United States Revisited
Source: Cancer Res Commun. 2024 Feb 7;4(2):328–36. doi: 10.1158/2767-9764.CRC-23-0503 (PMC10848893; doi:10.1158/2767-9764.CRC-23-0503)
Supplement: Supplementary Table 4 — shows sources for data. [file crc-23-0503-s04.pdf]

Supplementary Table 4: Reported Coefficients of Relative Position for Other Selected Cancers (with 95% Confidence Interval)

	Dependent variable:				
			Cancer Incidence Rate		
	Colon & Rectum	Liver & Bile Duct	Liver & Bile Duct (w/o MST)	Lung & Bronchus	Pancreas
	(1)	(2)	(3)	(4)	(5)
Relative Position	−0.011	−0.084**	−0.102***	0.140*	0.008
	(-0.098,0.077)	(-0.158,-0.010)	(-0.179,-0.025)	(-0.023,0.302)	(-0.038,0.055)
Latitude	0.203***	0.071	0.033	−0.025	0.132***
	(0.053,0.353)	(-0.067,0.208)	(-0.110,0.175)	(-0.306,0.257)	(0.054,0.210)
High School	21.027***	−5.935	−8.192	16.051	9.797***
	(9.925,32.128)	(-16.751,4.881)	(-19.274,2.890)	(-4.794,36.895)	(3.674,15.919)
Some College	−1.367	−1.847	−3.303	27.186***	7.266***
	(-10.666,7.932)	(-10.838,7.145)	(-12.572,5.966)	(9.601,44.770)	(2.119,12.413)
College and Above	−4.064	−13.262***	−16.222***	−22.630**	6.594**
	(-13.668,5.539)	(-22.371,-4.154)	(-25.542,-6.903)	(-40.985,-4.274)	(1.381,11.807)
Elevation	−0.001*	−0.001**	−0.002***	−0.009***	−0.0005
	(-0.002,0.0002)	(-0.002,-0.0002)	(-0.003,-0.001)	(-0.011,-0.007)	(-0.001,0.0002)
Medical Doctor pc	15.124	430.093***	422.909***	958.870***	73.399**
	(-104.990,135.238)	(318.346,541.841)	(309.565,536.252)	(711.023,1,206.717)	(7.949,138.849)
Median Income	0.00000	0.00000	0.00000	0.00001	−0.00000
	(-0.00001,0.00002)	(-0.00001,0.00002)	(-0.00001,0.00002)	(-0.00002,0.00004)	(-0.00001,0.00000)
Obesity Rate	−3.134	−4.150	−5.287*	30.056***	−1.452
	(-9.439,3.171)	(-10.068,1.768)	(-11.323,0.750)	(18.115,41.998)	(-4.903,1.998)
Smoking Rate	10.528	39.361***	35.483***	226.326***	8.799**
	(-5.842,26.897)	(24.681,54.041)	(20.469,50.498)	(194.047,258.606)	(0.133,17.465)
PM2.5 (air pollution)	0.004	0.018	0.013	0.346***	0.008
	(-0.118,0.127)	(-0.092,0.127)	(-0.099,0.125)	(0.115,0.577)	(-0.055,0.071)
Water Violation	−0.032	−0.344**	−0.320*	0.399	0.043
	(-0.399,0.335)	(-0.670,-0.018)	(-0.650,0.011)	(-0.325,1.122)	(-0.149,0.235)
Race (White)	−28.977*	−51.620***	−56.709***	−241.304***	−3.439
	(-58.255,0.300)	(-78.410,-24.830)	(-84.721,-28.697)	(-298.072,-184.536)	(-18.929,12.051)
Race (Black)	−23.891	−45.369***	−50.392***	−246.785***	0.912
	(-53.412,5.630)	(-72.414,-18.324)	(-78.647,-22.137)	(-304.098,-189.472)	(-14.714,16.538)
Race (Native)	−30.286*	−59.876***	−68.771***	−311.201***	−10.055
	(-61.974,1.403)	(-89.073,-30.678)	(-101.536,-36.006)	(-371.743,-250.659)	(-27.049,6.940)
Race (Asian)	−32.624**	−39.608***	−45.250***	−224.304***	−3.502
	(-63.812,-1.436)	(-68.239,-10.976)	(-75.110,-15.389)	(-284.614,-163.994)	(-20.001,12.996)
Race (Hispanic)	−30.277**	−43.964***	−50.357***	−259.359***	−2.417
	(-59.853,-0.701)	(-71.080,-16.847)	(-78.675,-22.040)	(-316.784,-201.934)	(-18.072,13.237)
Observations	1,955	1,117	1,063	2,441	1,090
R^2	0.418	0.556	0.541	0.823	0.334
Adjusted R^2	0.401	0.530	0.516	0.818	0.296
Residual Std. Error	0.318 (df = 1896)	0.404 (df = 1056)	0.404 (df = 1007)	0.431 (df = 2380)	0.265 (df = 1031)
F Statistic	23.511*** (df = 58; 1896)	21.996*** (df = 60; 1056)	21.576*** (df = 55; 1007)	184.027*** (df = 60; 2380)	8.909*** (df = 58; 1031)

Note: *p<0.1; **p<0.05; ***p<0.01
