# Supplementary material for: Longitudinal Position and Cancer Risk in the United States Revisited
Source: Cancer Res Commun. 2024 Feb 7;4(2):328–36. doi: 10.1158/2767-9764.CRC-23-0503 (PMC10848893; doi:10.1158/2767-9764.CRC-23-0503)
Supplement: Supplementary Table 5 — shows a Summary of Results Obtained by Gu et al. and Present Paper. [file crc-23-0503-s05.pdf]

Supplementary Table 5: Types of Cancer Included in Composite Cancer Incidence Rate (19 total)

| Cancer Type |
| --- |
| Bladder |
| Brain & ONS |
| Breast |
| Cervix |
| Colon & rectum |
| Esophagus |
| Kidney & renal pelvis |
| Leukemia |
| Liver & bile duct |
| Lung & bronchus |
| Melanoma of the skin |
| Non-hodgkin lymphoma |
| Oral cavity & pharynx |
| Ovary |
| Pancreas |
| Prostate |
| Stomach |
| Thyroid |
| Uterus |
