# Supplementary material for: Longitudinal Position and Cancer Risk in the United States Revisited
Source: Cancer Res Commun. 2024 Feb 7;4(2):328–36. doi: 10.1158/2767-9764.CRC-23-0503 (PMC10848893; doi:10.1158/2767-9764.CRC-23-0503)
Supplement: Supplementary Table 6 — shows Reported Coefficients of Relative Position for Hormonally Associated Cancers (with 95% Confidence Interval) [file crc-23-0503-s06.pdf]

Supplementary Table 6: Counties and Cities combined in Virginia

County + City
Albemarle + Charlottesville
Alleghany + Covington
Augusta, Staunton + Waynesboro
Campbell + Lynchburg
Carroll + Galax
Dinwiddie, Colonial Heights + Petersburg
Fairfax, Fairfax City + Falls Church
Frederick + Winchester
Greensville + Emporia
Henry + Martinsville
James City + Williamsburg
Montgomery + Radford
Pittsylvania + Danville
Prince George + Hopewell
Prince William, Manassas + Manassas Park
Roanoke + Salem
Rockbridge, Buena Vista + Lexington
Rockingham + Harrisonburg
Southampton + Franklin
Spotsylvania + Fredericksburg
Washington + Bristol
Wise + Norton
York + Poquoson
