# Supplementary material for: Longitudinal Position and Cancer Risk in the United States Revisited
Source: Cancer Res Commun. 2024 Feb 7;4(2):328–36. doi: 10.1158/2767-9764.CRC-23-0503 (PMC10848893; doi:10.1158/2767-9764.CRC-23-0503)
Supplement: Supplementary Table 7 — shows Reported Coefficients of Relative Position for Other Selected Cancers (with 95% Confidence Interval) [file crc-23-0503-s07.pdf]

Supplementary Table 7: Summary Table of Results Obtained by Gu et al. and Present Paper

Topic	Findings of Gu et al.	Findings of this paper
Number of observations for composite cancer rate (counties)	607	2853
Race and ethnicity studied	White	White, black, American Indian / AK native, Asian / Pacific Islander, Hispanic (any race)
Composite cancer (inconsistent)	RR* = 1.029 (significant**)	RR_eqv*** = 2.000
Breast cancer (consistent)	RR = 1.074	RR_eqv = 1.425 (significant)
Ovary cancer	—	RR_eqv = 0.030
Prostate cancer (inconsistent)	RR = 1.042 (significant)	RR_eqv = -1.935 (significant)
Thyroid cancer	RR = 1.042	RR_eqv = -0.615
Liver and bile duct cancer (consistent)	RR = 1.110 (significant)	RR_eqv = 0.510 (significant)
Lung and bronchus cancer	RR = 1.002	RR_eqv = -0.700
Colon and rectum cancer	RR = 1.023	RR_eqv = 0.055
Pancreas cancer	RR = 1.041	RR_eqv = -0.040

* Response variable used by Gu et al. is covariate-adjusted rate ratio (RR) per 5 degrees difference in longitude, which is equivalent to five times the regression coefficient of our model in magnitude.
Furthermore, they interpret their RR as "moving from the east to the west" while we interpret our coefficient as moving from the west to the east. Therefore, we also flip the sign of their RR in order to make the comparison with our estimated coefficients.
** All significance represents the 0.01 significance level.
*** RR_eqv is derived from the coefficients in our model through the progress described above (namely RR_eqv = -coef*5).
