# Supplementary material for: Longitudinal Position and Cancer Risk in the United States Revisited
Source: Cancer Res Commun. 2024 Feb 7;4(2):328–36. doi: 10.1158/2767-9764.CRC-23-0503 (PMC10848893; doi:10.1158/2767-9764.CRC-23-0503)
Supplement: Supplementary Figure 1 — shows a map of U.S. counties contained in our analysis of composite cancer incidence according to their respective time zones (n = 2853). [file crc-23-0503-s08.pdf]

1 Supplementary Information

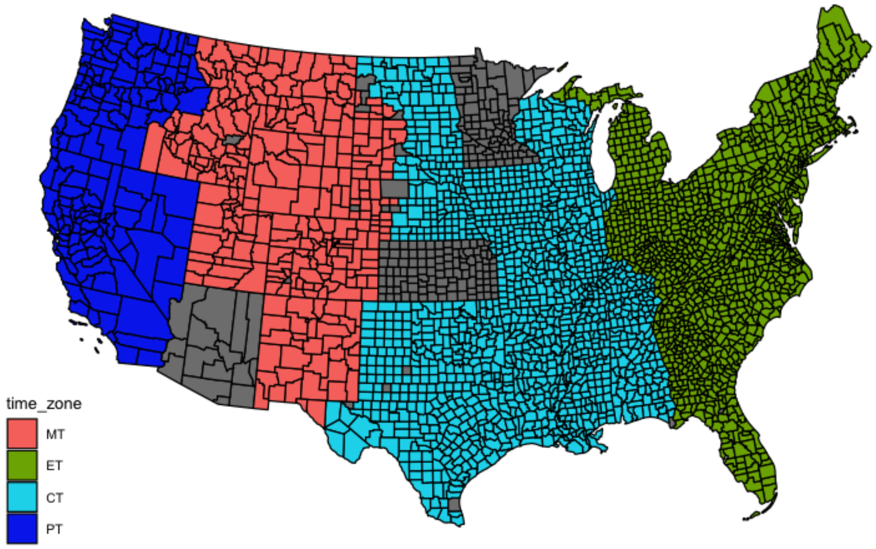

Supplementary Figure 1: U.S. Counties By Time Zone ($n = 2853$)
Supplementary Figure 1 shows a map of U.S. counties contained in our analysis of composite cancer incidence according to their respective time zones ($n = 2853$).
