# Supplementary material for: Longitudinal Position and Cancer Risk in the United States Revisited
Source: Cancer Res Commun. 2024 Feb 7;4(2):328–36. doi: 10.1158/2767-9764.CRC-23-0503 (PMC10848893; doi:10.1158/2767-9764.CRC-23-0503)
Supplement: Supplementary Figure 3 — shows maps of cancer incidence rate by county for most prevalent cancer. [file crc-23-0503-s10.pdf]

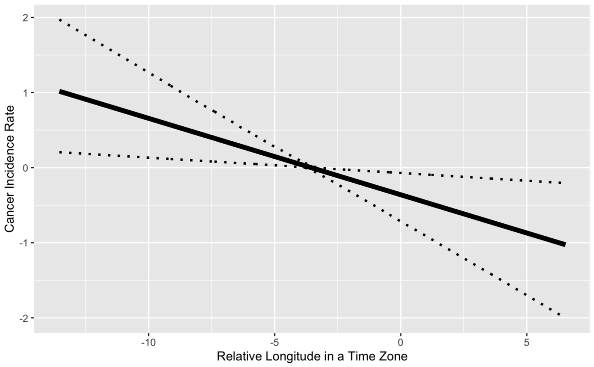

(a) Liver & Bile Duct Cancer (without MST, $n = 1063$)

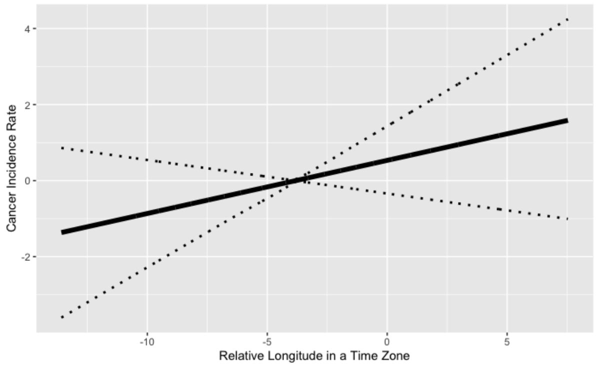

(b) Lung & Bronchus Cancer ($n = 2441$)

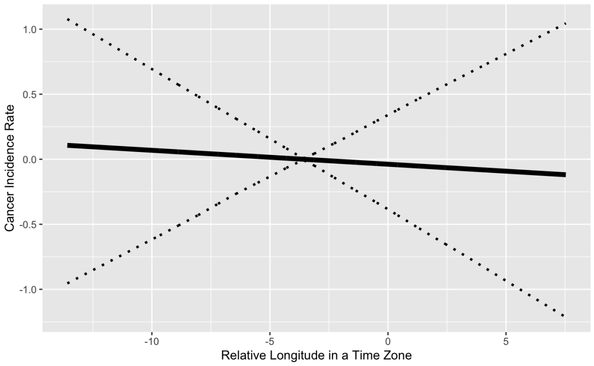

(c) Colon & Rectum Cancer ($n = 1955$)

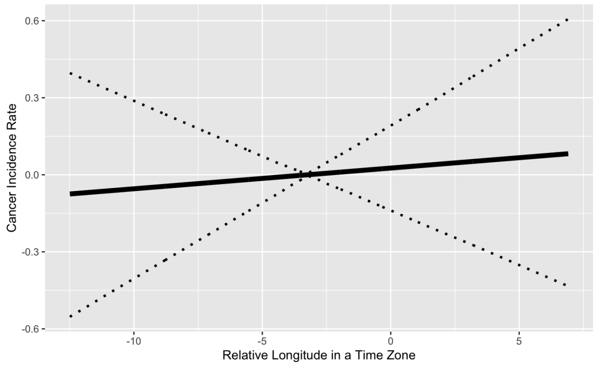

(d) Pancreas Cancer ($n = 1191$)

Supplementary Figure 3: Output of Linear Approximation for Most Prevalent Cancer Incidence Supplementary Figure 3 shows the linear approximation result of incidence by relative position for four of the most prevalent cancers, with 95% bootstrap confidence band.
