# Supplementary material for: Longitudinal Position and Cancer Risk in the United States Revisited
Source: Cancer Res Commun. 2024 Feb 7;4(2):328–36. doi: 10.1158/2767-9764.CRC-23-0503 (PMC10848893; doi:10.1158/2767-9764.CRC-23-0503)
Supplement: Supplementary Figure 4 — shows the relative weights of 607 counties studied by Gu et al. (2017) [file crc-23-0503-s11.pdf]

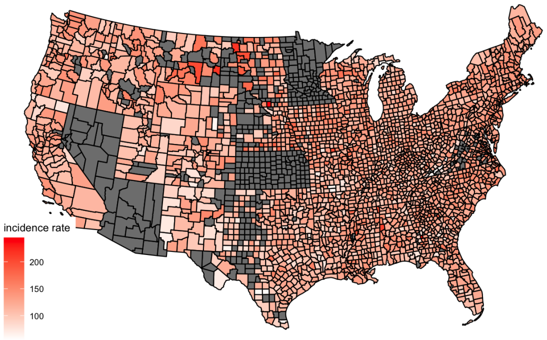

(a) Breast Cancer ($n = 2615$)

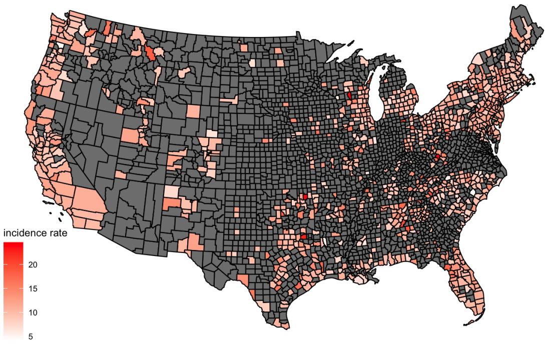

(b) Ovary Cancer ($n = 890$)

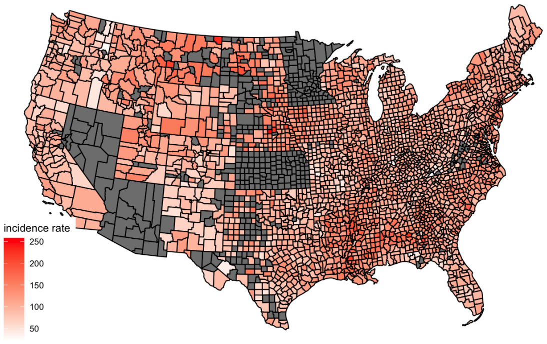

(c) Prostate Cancer ($n = 2623$)

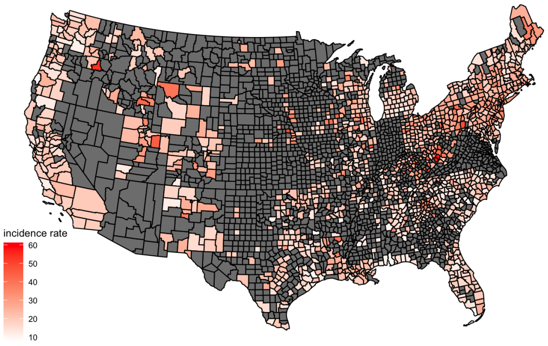

(d) Thyroid Cancer ($n = 1191$)

Supplementary Figure 4: U.S. Map of Cancer Incidence Rate for Hormonally Associated Cancers
Supplementary Figure 4 shows maps of cancer incidence rate by county for four of the hormonally associated cancers.
