# Supplementary material for: Longitudinal Position and Cancer Risk in the United States Revisited
Source: Cancer Res Commun. 2024 Feb 7;4(2):328–36. doi: 10.1158/2767-9764.CRC-23-0503 (PMC10848893; doi:10.1158/2767-9764.CRC-23-0503)
Supplement: Supplementary Figure 5 — shows the output of natural splines conducted on incidence by longitude and time zone for composite cancer with 95% bootstrap confidence band using linear approximation (n = 2853). [file crc-23-0503-s12.pdf]

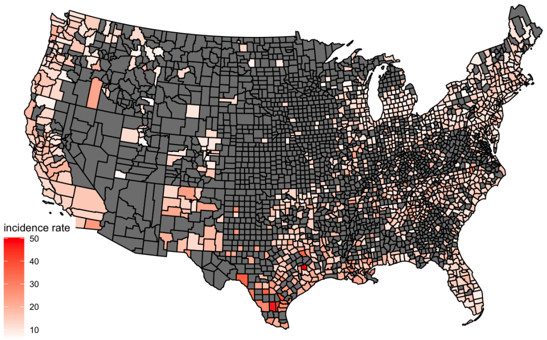

(a) Liver & Bile Duct Cancer ($n = 1117$)

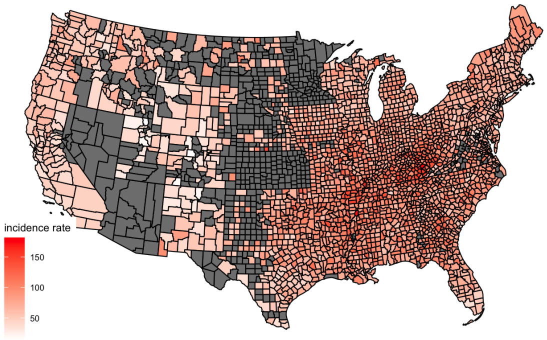

(b) Lung & Bronchus Cancer ($n = 2441$)

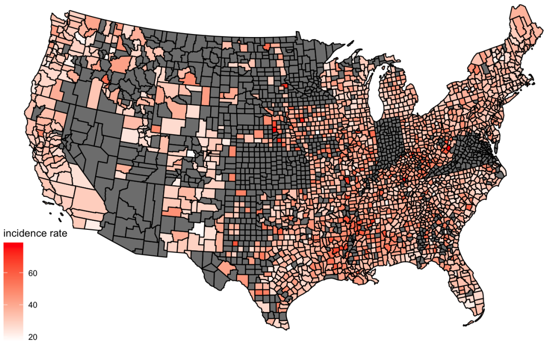

(c) Colon & Rectum Cancer ($n = 1955$)

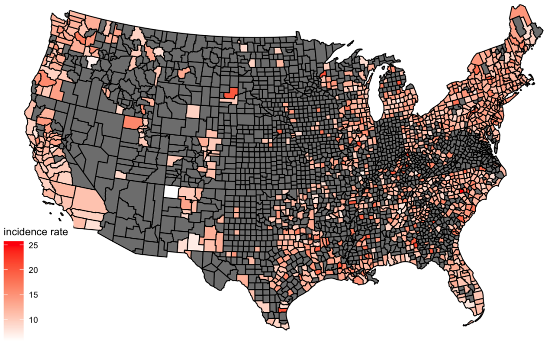

(d) Pancreas Cancer ($n = 1191$)

Supplementary Figure 5: U.S. Map of Cancer Incidence Rate for Four Most Prevalent Cancers
Supplementary Figure 5 shows maps of cancer incidence rate by county for most prevalent cancer.
