# Supplementary material for: Longitudinal Position and Cancer Risk in the United States Revisited
Source: Cancer Res Commun. 2024 Feb 7;4(2):328–36. doi: 10.1158/2767-9764.CRC-23-0503 (PMC10848893; doi:10.1158/2767-9764.CRC-23-0503)
Supplement: Supplementary Figure 6 — shows the linear approximation result of incidence by relative position for four of the hormonally associated cancers, with 95% bootstrap confidence band. [file crc-23-0503-s13.pdf]

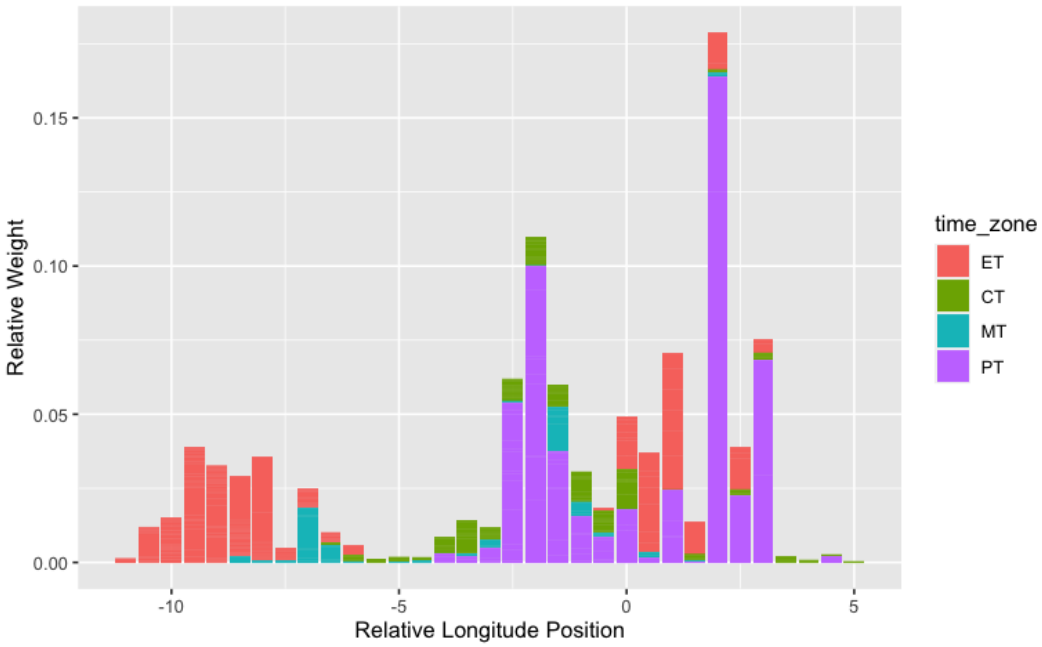

Supplementary Figure 6: Relative Weights of 607 Counties studied by Gu et al. (2017)
Supplementary Figure 6 shows the relative weights of 607 counties studied by Gu et al. (2017)
