# Supplementary material for: Longitudinal Position and Cancer Risk in the United States Revisited
Source: Cancer Res Commun. 2024 Feb 7;4(2):328–36. doi: 10.1158/2767-9764.CRC-23-0503 (PMC10848893; doi:10.1158/2767-9764.CRC-23-0503)
Supplement: Supplementary Figure 7 — shows the linear approximation result of incidence by relative position for four of the most prevalent cancers, with 95% bootstrap confidence band. [file crc-23-0503-s14.pdf]

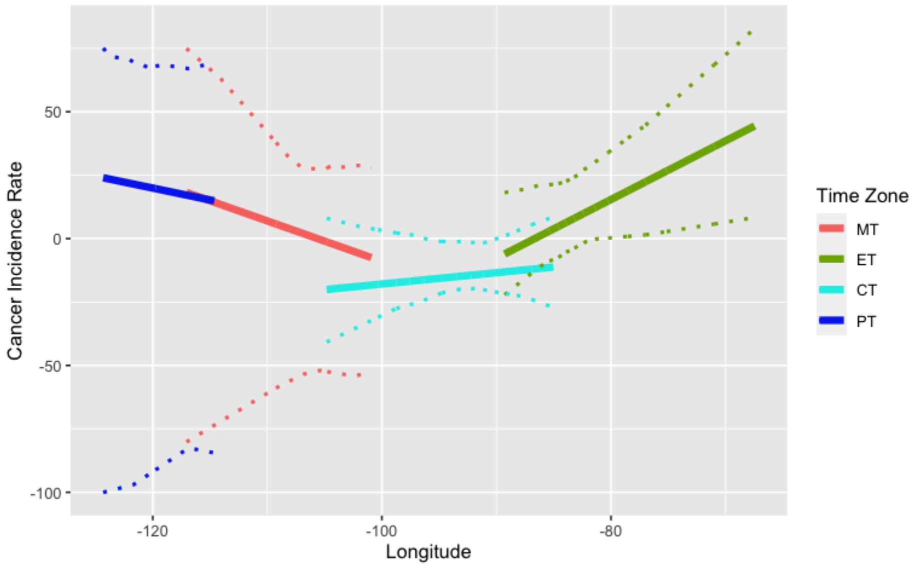

Supplementary Figure 7: Output of Natural Splines for Composite Cancer Incidence with Linear Approximation Supplementary Figure 7 shows the output of natural splines conducted on incidence by longitude and time zone for composite cancer with 95% bootstrap confidence band using linear approximation (n = 2853)
